# Comprehensive identification and expression analyses of the *SnRK* gene family in *Casuarina equisetifolia* in response to salt stress

**DOI:** 10.1186/s12870-022-03961-7

**Published:** 2022-12-09

**Authors:** Di Ai, Yujiao Wang, Yongcheng Wei, Jie Zhang, Jingxiang Meng, Yong Zhang

**Affiliations:** 1 Research Institute of Tropical Forestry, Chinese Academy of Forestry, Guangzhou, 510520 China; 2 College of Landscape Architecture, Northeast Forestry University, Harbin, China

**Keywords:** Phylogenetic analysis, *Cis*-regulatory element analysis, Expression pattern, Salt stress

## Abstract

**Background:**

Sucrose nonfermenting-1 (SNF1)-related protein kinases (SnRKs) play crucial roles in plant signaling pathways and stress adaptive responses by activating protein phosphorylation pathways. However, there have been no comprehensive studies of the *SnRK* gene family in the widely planted salt-tolerant tree species *Casuarina equisetifolia*. Here, we comprehensively analyze this gene family in *C. equisetifolia* using genome-wide identification, characterization, and profiling of expression changes in response to salt stress.

**Results:**

A total of 26 *CeqSnRK* genes were identified, which were divided into three subfamilies (SnRK1, SnRK2, and SnRK3). The intron–exon structures and protein‑motif compositions were similar within each subgroup but differed among groups. Ka/Ks ratio analysis indicated that the *CeqSnRK* family has undergone purifying selection, and cis-regulatory element analysis suggested that these genes may be involved in plant development and responses to various environmental stresses. A heat map was generated using quantitative real‑time PCR (RT-qPCR) data from 26 *CeqSnRK* genes, suggesting that they were expressed in different tissues. We also examined the expression of all *CeqSnRK* genes under exposure to different salt concentrations using RT-qPCR, finding that most *CeqSnRK* genes were regulated by different salt treatments. Moreover, co-expression network analysis revealed synergistic effects among *CeqSnRK* genes.

**Conclusions:**

Several *CeqSnRK* genes (*CeqSnRK3.7, CeqSnRK3.16, CeqSnRK3.17*) were up-regulated following salt treatment. Among them, *CeqSnRK3.16* expression was significantly up-regulated under various salt treatments, identifying this as a candidate gene salt stress tolerance gene. In addition, *CeqSnRK3.16* showed significant expression change correlations with multiple genes under salt stress, indicating that it might exhibit synergistic effects with other genes in response to salt stress. This comprehensive analysis will provide a theoretical reference for *CeqSnRK* gene functional verification and the role of these genes in salt tolerance.

**Supplementary Information:**

The online version contains supplementary material available at 10.1186/s12870-022-03961-7.

## Background

Salinity, due mainly to sodium chloride (NaCl), seriously affects plant growth and development. The presence of redundant salt ions causes damage by inducing oxidation stress, osmotic stress, and ion toxicity [1, 2]. *Casuarina equisetifolia* is a sort of salt-resistant tree widely planted in southern China in coastal shelterbelts to stabilize moving sands, provide fuel wood, and reclaim coastal ecosystems due to its superior biological characteristics, such as rapid growth, wind and salt tolerance, and nitrogen fixation [3–5]. Therefore, it has become the most important shelterbelt tree species in this coastal area. According to a previous study, *C. equisetifolia* is highly salt tolerant and can survive in 500 mM NaCl solution [6]. It would therefore be useful to identify the determinants of salt tolerance in this species.

In general, plants respond to adverse environmental pressure, including salt stress, in two methods: regulation of gene expression and modification of proteins [7]. Among them, protein kinase-mediated phosphorylation and dephosphorylation is one way of protein modification [8]. SnRKs (sucrose nonfermenting 1 (SNF1)-related protein kinases) are Ser/Thr protein kinases involved in various physiological activities [9, 10]. The *SnRK* gene family was segmented into three subfamilies, SnRK1, SnRK2, and SnRK3 [11, 12]. The SnRK1 subfamily shares a strong identity due to an extremely conservative N-terminal catalytic domain [13, 14]. In contrast to the SnRK1 subfamily, the SnRK2 and SnRK3 subfamilies are plant-specific and more diverse [15, 16]. Apart from the identical kinase domain at the N-terminus, each SnRK2 subfamily member contains a C-terminal diverse regulatory domain and an adenosine triphosphate (ATP) binding domain [17]. SnRK3s, also referred to as calcineurin B-like protein-interacting protein kinases (CIPKs), interact with and regulate calcineurin B-like protein (CBL) for transient decoding of calcium signals [18–20]. They possess two conserved domains, NAF and PPI, at the C terminus [21].

Previous studies had shown that *SnRK* genes could make a response to manifold stresses, of which salt stress was an important one [14, 17, 22]. Over-expression of *TaSnRK2.9* (*Triticum aestivum*) in tobacco (*Nicotiana tabacum*) plants increases scavenging of reactive oxygen species (ROS), thereby promoting the maintenance of ROS at a normal level to protect against abiotic stress [23]. The over-expression of *TaSnRK2.3*/-*2.4*/*-2.7*/-*2.8* in *Arabidopsis thaliana* had been reported to heighten plant resistance to salt and other stresses [24–26]. In addition, studies had confirmed that *PtSnRK2.5* and *PtSnRK2.7* in *Populus trichocarpa* were closely related to the salt tolerance of transgenic *A. thaliana* [27]. Similarly, *BdSnRK2.9* in *Brachypodium distachyon* was shown to increase the tolerance of transgenic tobacco to high NaCl concentration treatment [28], and *ZmCIPK21* and *OsCIPK15* were found could improve plant resistance to salt stress in maize (*Zea mays*) and rice (*Oryza sativa*), respectively [29, 30]. Moreover, SnRK3s participates in the SOS (salt overly sensitive) stress signal transduction pathway to regulate intracellular ion homeostasis [31]. For example, the calcium signal generated by salt stress could be intelligently recognized by SOS3 (AtCBL4), and then eliminated superfluous Na^+^ from root cells by co-phosphorylating SOS1 (Na^+^/H^+^ antitransporter) with SOS2 (AtCIPK24) [32]. *AtCIPK24* could bind to the photoperiod and circadian clock regulator GI (GIGANTEA), further inhibiting the *SOS1* signaling process to regulate the adaptability of plants to salinity [33]. In summary, *SnRK* genes are important in salt stress responses, and their genetic modification could potentially improve plant salt tolerance. However, a functional understanding of *SnRK* genes in *C. equisetifolia* has been lacking, leaving it unclear what role(s) *SnRK* genes might play in this species.

In our study, a total of 26 *SnRK* gene family members were identified in the genome of *C. equisetifolia*. We then evaluated their phylogenetic relationships, physicochemical property, gene structure, conserved domains, and promoter analysis using bioinformatics. Furthermore, differential expression patterns of *CeqSnRK* genes in different tissues and under various salt treatments were profiled using RT-qPCR. These results lay a substantial groundwork for further investigations of the molecular mechanism in *C. equisetifolia* resistance to salt stress.

## Results

### Identification of SnRK genes in C. equisetifolia

As confirmed by SMART, Pfam, and manual screening, twenty-six *SnRK* proteins in *C. equisetifolia* were identified. According to the nomenclature in *A. thaliana*, we named these *CeqSnRK1.1* to *CeqSnRK1.2*, *CeqSnRK2.1* to *CeqSnRK2.7*, and *CeqSnRK3.1* to *CeqSnRK3.17*. These 26 *CeqSnRK* genes were distributed across 21 scaffolds (the genome of *C. equisetifolia* is presently assembled to the scaffold level). Their coding sequences ranged from 247 (*CeqSnRK2.4*) to 541 amino acids (*CeqSnRK3.7*) in length. Moreover, the molecular weight and pI ranged from 27.71 kDa (*CeqSnRK2.4*) to 61.48 kDa (*CeqSnRK3.7*) and from 4.43 (*CeqSnRK2.4*) to 9.21 (*CeqSnRK3.3*), respectively. The detailed parameters were presented in Table S1. The results of subcellular localization prediction showed that the SnRK1 subfamily and the majority of SnRK2 subfamily proteins were situated in the cytoplasm and nucleus, but *CeqSnRK2.1*, *CeqSnRK2.2*, *CeqSnRK2.5*, and *CeqSnRK2.6* were located in the cytoskeleton. The SnRK3 subfamily members were mostly located in the cytoplasm and chloroplast, while *CeqSnRK3.2* was located in the endoplasmic reticulum. The details were provided in Table S2.

### Phylogenetic tree and multiple alignment of CeqSnRK genes

**Fig. 1:**
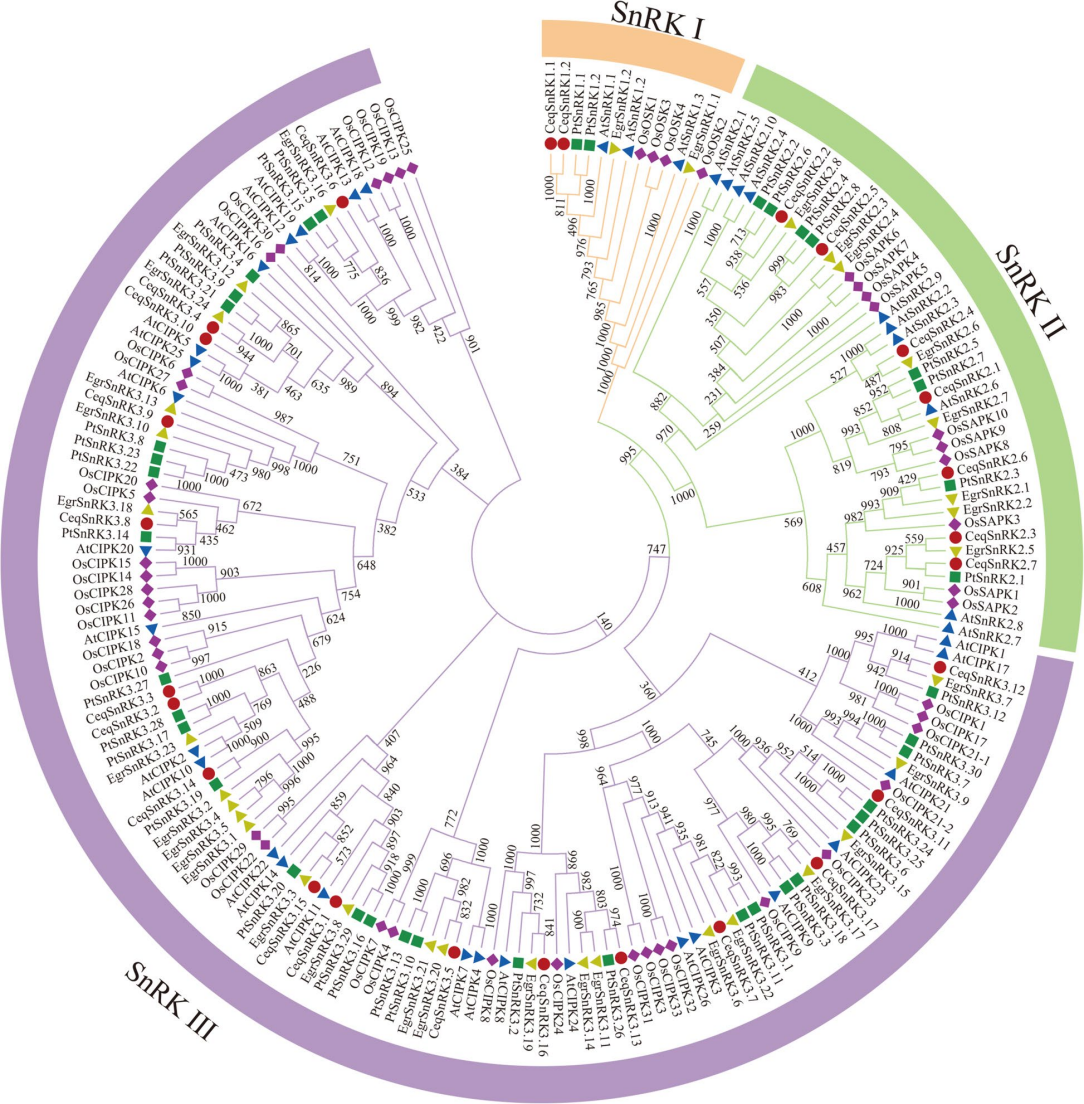
Phylogenetic tree of *SnRK* genes from *Casuarina equisetifolia*, *Arabidopsis thaliana*, *Populous trichocarpa*, *Eucalyptus grandis*, and *Oryza sativa*. A total of 26 *CeqSnRK* genes, 34 *EgrSnRK* genes, 39 *AtSnRK* genes, 45 *PtSnRK* genes and 48 *OsSnRK* genes were clustered into three subgroups (SnRK1, SnRK2 and SnRK3). Details of the *SnRK* genes from all plant species are listed in Table S2. The tree was generated using ClustalX 2.0 with the neighbor-joining (N-J) method

To reveal the phylogenetic relation of SnRK proteins from *A. thaliana*, *Eucalyptus grandis*, *P. trichocarpa*, *Oryza sativa*, and *C. equisetifolia*, we then built a phylogenetic tree using MEGA 7.0 with the neighbor-joining (NJ) method (Fig. 1). Additionally, detailed information on the protein sequences of the above-mentioned plants can be found in Table S3. The 186 SnRK proteins were distinctly split into three subfamilies: SnRK1, SnRK2, and SnRK3. The phylogenetic analysis indicated that the SnRK1 subfamily had the smallest and the SnRK3 subfamily contained the largest number of genes. In addition, these *SnRK* genes in each subfamily was even-distributed, except that the rice *SnRK* genes showed clear clustering, which might be caused by differences between monocotyledons and dicotyledons.

**Fig. 2:**
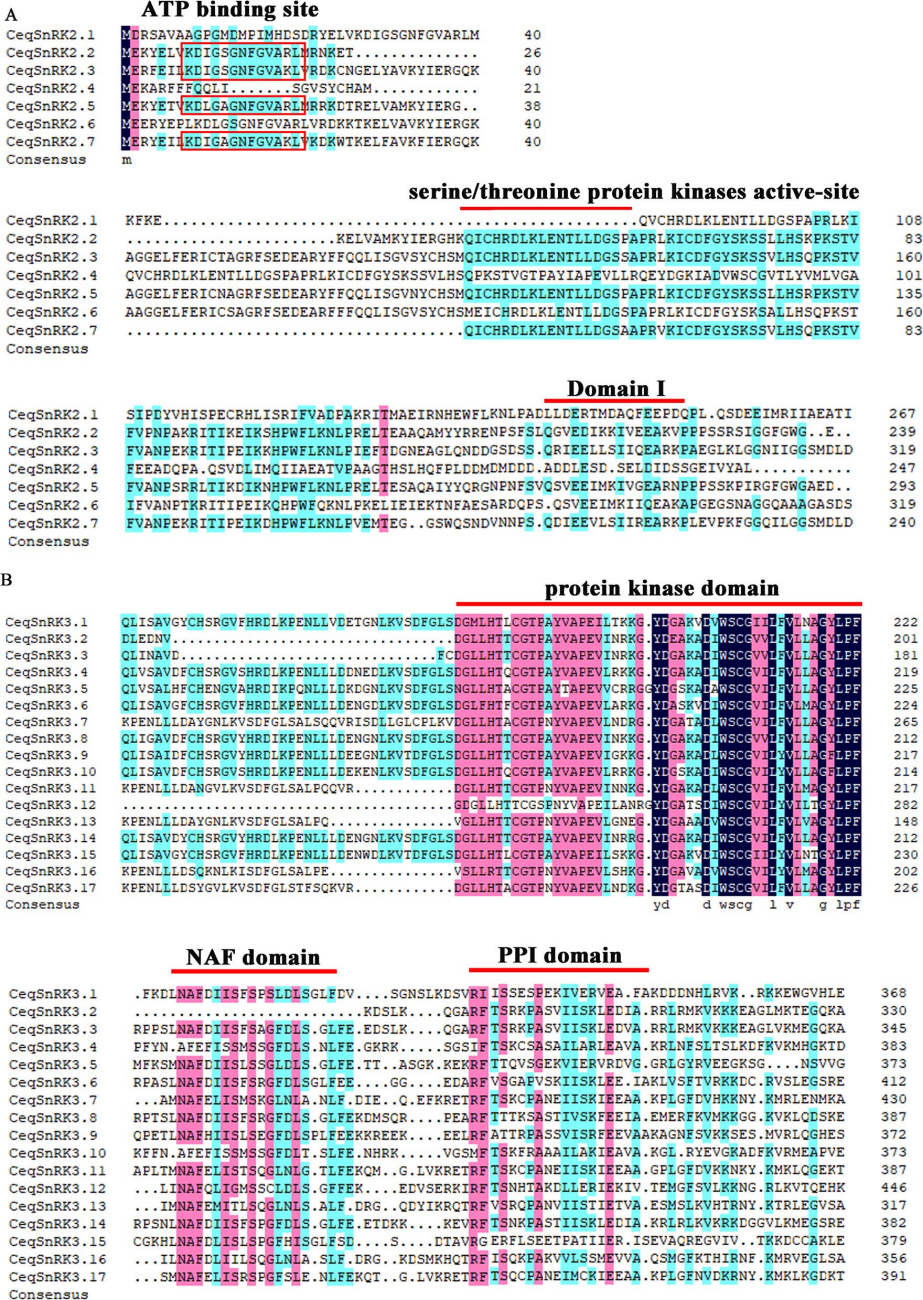
**A** Multiple sequence alignment of *CeqSnRK2* genes. **B** Multiple sequence alignment of *CeqSnRK3* genes. Sequences were aligned using DNAMAN 8.0. The scale indicates the number of amino acids. Blue and red indicate conserved amino acids. Red lines and boxes indicate conserved domains

The multiple sequence alignment of the *CeqSnRK*s was further probed using DNAMAN 8.0 software. Results showed that all of *CeqSnRK2* genes encoded an ATP binding site and serine/threonine protein kinase active site at N-terminal regions and a domain known as domain I at the C terminus that is indispensable for enhancing osmotic stress–mediated endurance (Fig. 2). *CeqSnRK3* genes not only contained a protein kinase domain at the N terminus, but specifically encoded a NAF domain and PPI domain at the C terminus. In summary, the multiple sequence alignment validated that the *CeqSnRK* genes have complete functional domains.

### Gene structure, motif composition, and protein structural analyses

**Fig. 3:**
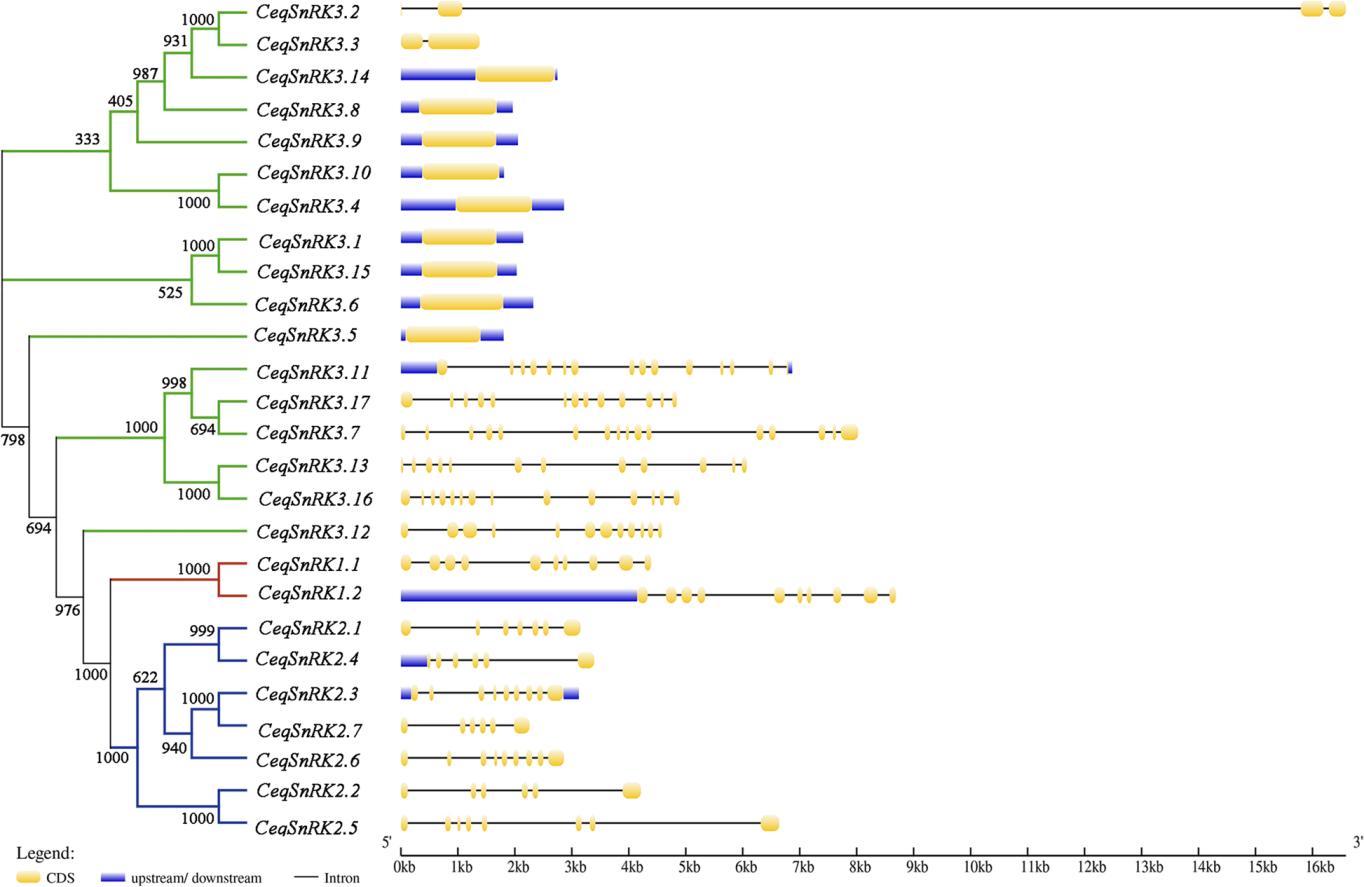
Phylogenetic relationships and gene structure of *SnRK* genes in *Casuarina equisetifolia*. *Left panel* shows the phylogenetic tree of 26 *CeqSnRK* genes built using the neighbor-joining method. Red, green, and blue indicate the SnRK1, SnRK2, and SnRK3 subgroups, respectively. *Right panel* shows the gene structure of *CeqSnRK* genes. Exons are indicated by yellow rectangles. Gray lines connecting two exons represent introns

The exon–intron structures of the 26 *CeqSnRK* genes were analyzed to facilitate understanding of gene evolution (Fig. 3). It turned out that the genetic structures of members of the same subfamily shared analogical characteristics. Members of the CeqSnRK1 subfamily had nine introns, consistent with those of *E. grandis* [34], while the number of introns in the CeqSnRK2 subfamily changed from five to eight. However, there was a noticeable difference in the intron numbers of CeqSnRK3 subfamily members, and they were segmented into two forms: intron-free and intron-rich. Nine *CeqSnRK3* genes had no introns, 6 *CeqSnRK3* genes had 11 to 15 introns, and the remaining two genes, *CeqSnRK3.2* and *CeqSnRK3.3*, had either 3 or 1 intron (Fig. 3).

**Fig. 4:**
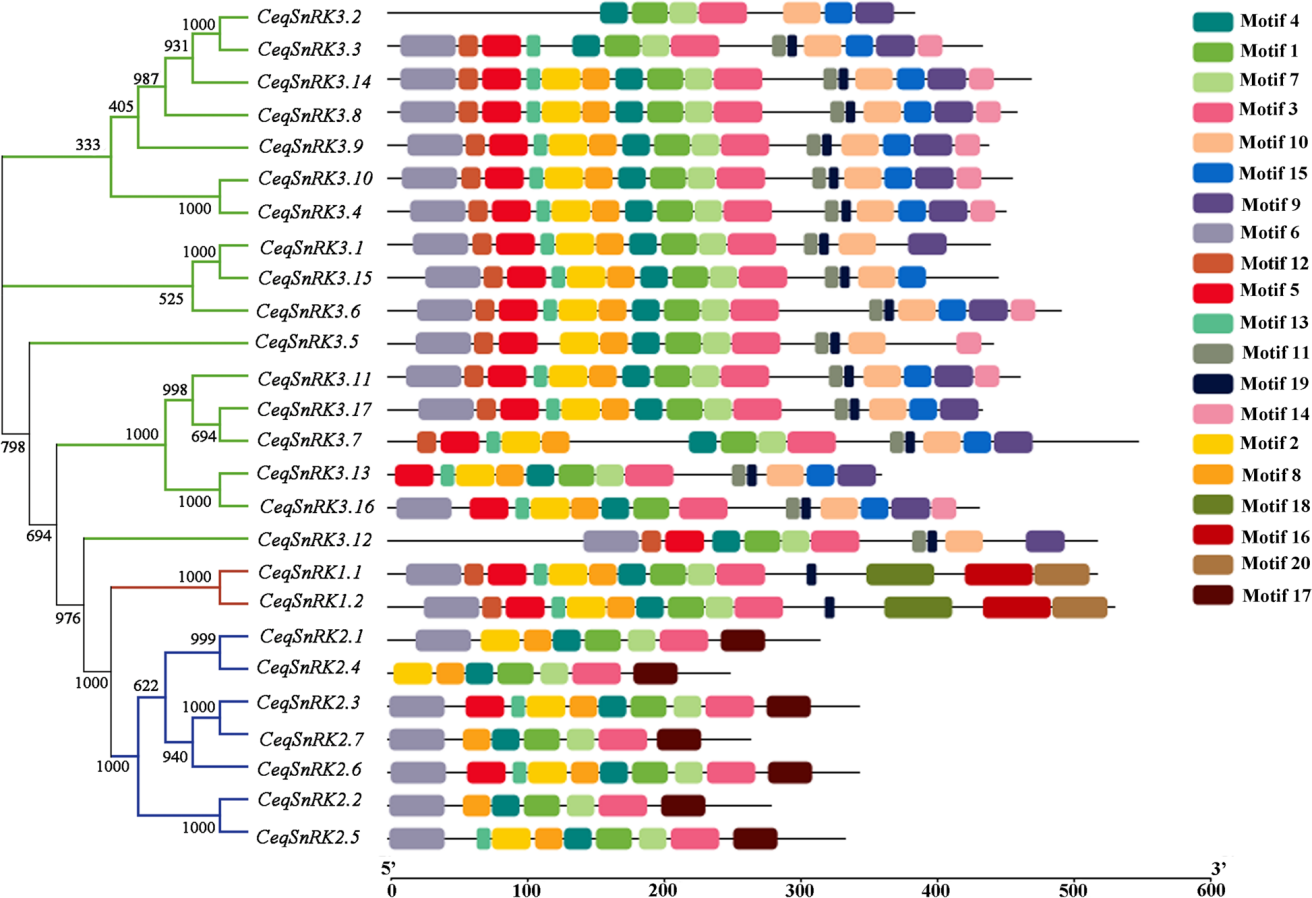
Conserved motifs of *SnRK* genes in *Casuarina equisetifolia*. The distribution of 20 conserved motifs in *CeqSnRK* genes was analyzed using MEME. Rectangles with different colors represent different motifs

**Fig. 5:**
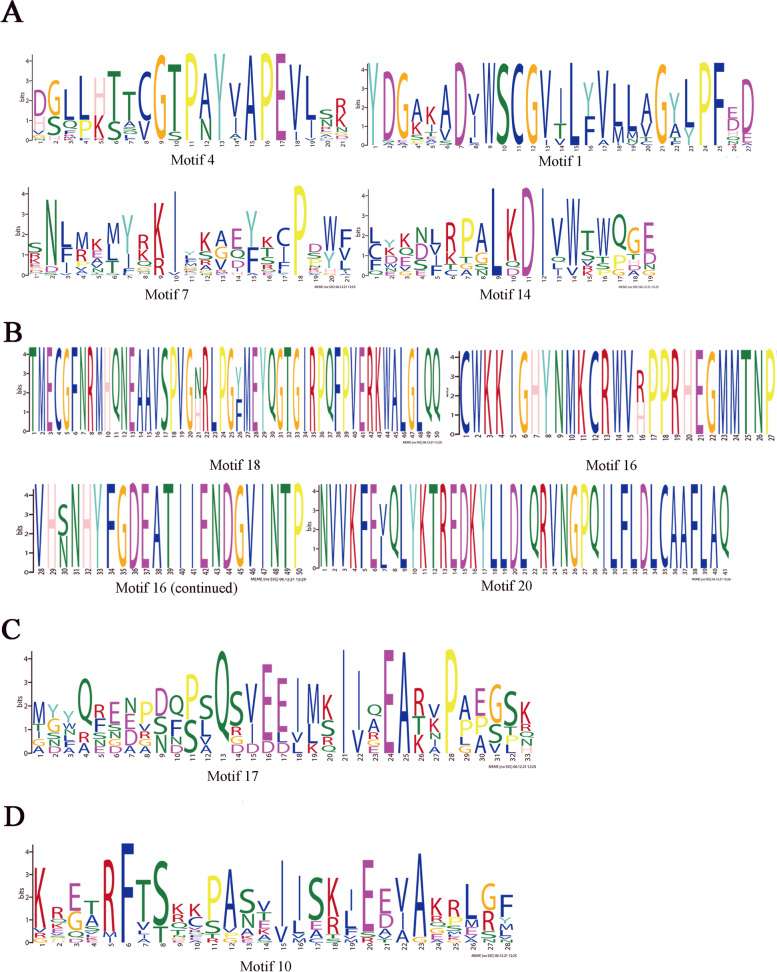
Sequence logos of repeated domains in the *CeqSnRK* gene family. **A** Motifs shared by SnRK1, SnRK2, and SnRK3 subfamilies. **B** Motifs unique to the SnRK1 subfamily. **C** Motif unique to the SnRK2 subfamily. **D** Motif unique to the SnRK3 subfamily. The overall height of each stack shows the conservation of the *SnRK* protein sequence at that position. Letters indicate different amino acid residue

To further reveal the structure of *CeqSnRK* proteins, we identified 20 conserved motifs and assessed their distribution (Fig. 4); the details were shown in Table S4. According to the Pfam annotation results, motifs 1, 2, and 3 encode a protein kinase domain; motifs 10 and 11 encode a NAF domain; and motif 20 encodes a KA1 domain. The other motifs had no functional annotations based on the Pfam database. We then used WebLogo and found that four motifs (motifs 1, 4, 7, and 14) were present in all of the *CeqSnRK* members (Fig. 5A); motifs 16, 18, and 20 were present in all members of the *CeqSnRK1* subfamily (Fig. 5B); and motifs 17 and 10 were unique to the *CeqSnRK2* and *CeqSnRK3* subfamilies, respectively (Fig. 5C, D). In conclusion, the motifs in *CeqSnRK* genes of the same subfamily contained similar regularity, indicating that they might share the same genetic structure and functional domains.

The functions of a protein are closely related with its structure; therefore, the secondary and tertiary protein structures of each *CeqSnRK* gene were analyzed. We predicted the secondary structures of *CeqSnRK* genes using the Phyre 2 software, finding that alpha helix and random coil accounted for a major proportion (Table S5). Furthermore, 3D models of *CeqSnRK* genes were constructed using Swiss-Model online server (Fig. S1). As illustrated in the Fig. S1, the 3D structures of *CeqSnRK*s were variable among different subfamilies, indicating the existence of potential functional diversity.

### Homology analysis in C. equisetifolia

**Fig. 6:**
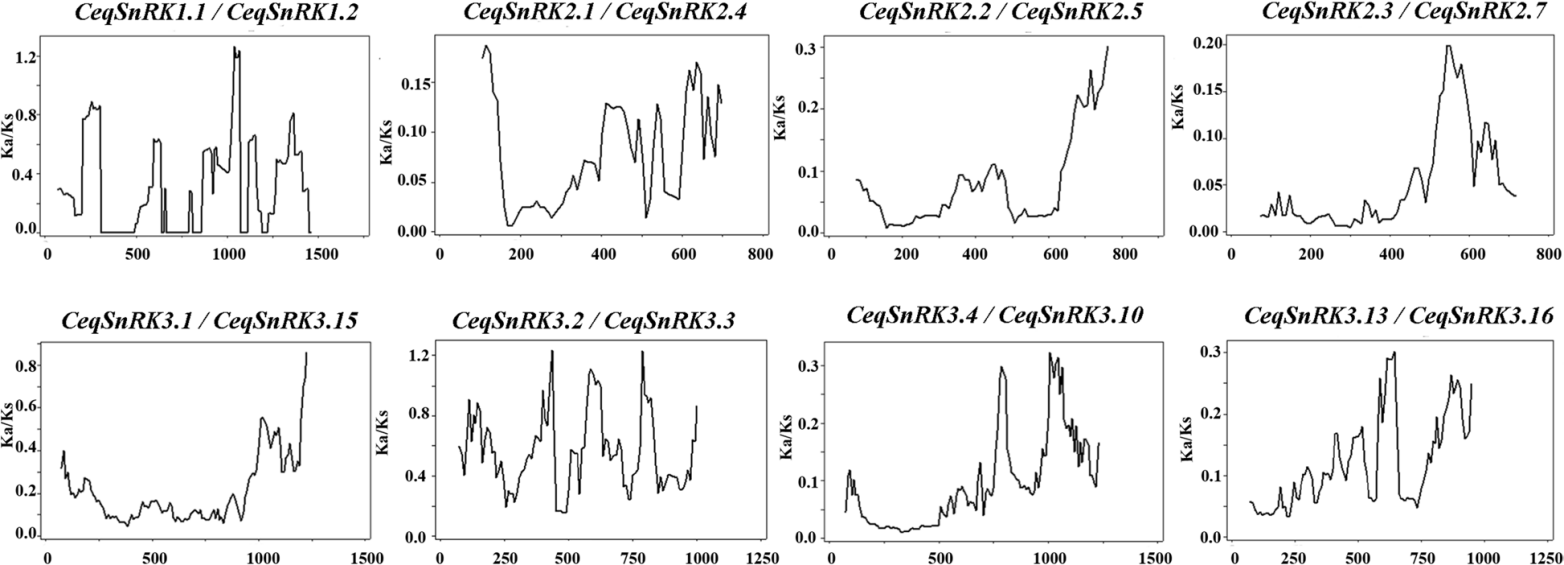
Sliding-window analysis of eight paralogous gene pairs. Window size, 150 bp; step size, 9 bp

We identified 43 *SnRK* orthologues and 8 paralogues on the basis of the topology of the phylogenetic tree and BLASTN results. To further explore the effect of selection pressure on the evolution of *CeqSnRK* genes, the synonymous substitutions (Ks), non-synonymous substitutions (Ka), and the Ka*/*Ks ratios of paralogues and orthologues were calculated using DnaSP 5.0 software. We built a sliding-window analysis for paralogous genes (Fig. 6); the Ka*/*Ks values of the orthologues are shown in Fig. S2. The ratio of Ka/Ks can be used to judge the selective pressure. Generally, when the ratio is equal to 1, it means neutral selection, intimating that the DNA mutation exerts no influence on the organism. Ka/Ks < 1 indicates purification selection (negative selection), while Ka/Ks > 1 indicates accelerated evolution (positive selection) [27]. We discovered that the Ka/Ks ratios of the paralogue pairs in *C. equisetifolia* ranged from 0.037 to 0.751 (Table S6), indicating that the *CeqSnRK* gene family has undergone purifying selection.

### Promoter analyses

**Fig. 7:**
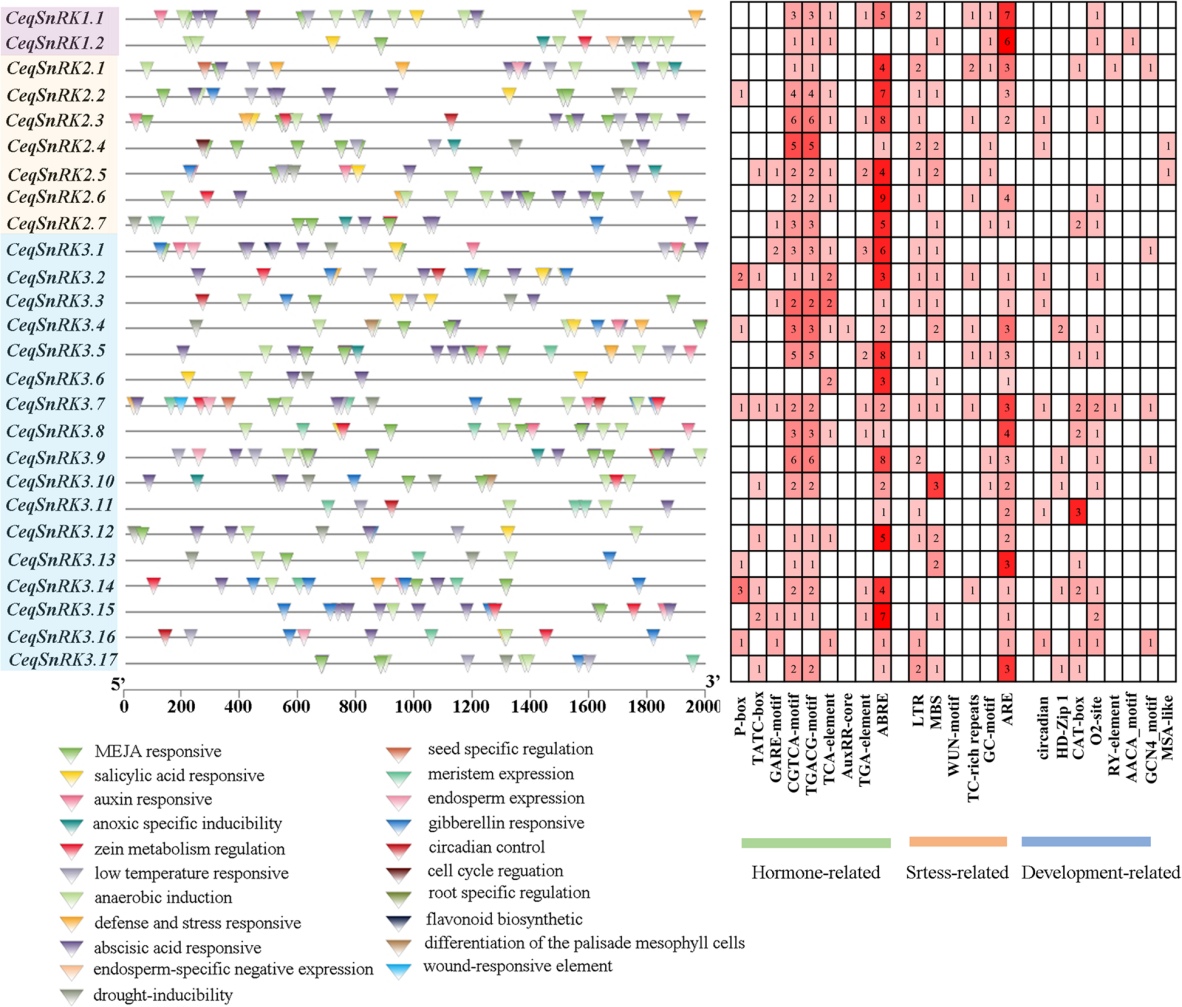
Analysis of cis-acting elements in the promoter regions of *CeqSnRK* genes. Binding sites in the promoter region are represented by boxes of different colors, and the graph shows the number of binding sites

In order to determine the cis-acting elements of the *CeqSnRK* genes, the promoter region of the *CeqSnRK* gene (the genomic DNA sequence 2 kb upstream of the translation start point) was submitted to the PlantCARE database for search, which will be conducive to further understanding of gene function and regulation (Table S7). The results identified three classes of cis-elements associated with stress responses, hormone responses, and plant development. In Fig. 7, a total of 98 ABA-responsive elements (ABRE) were found in the promoters of 24 *CeqSnRK* genes, of which *CeqSnRK2* genes all contained ABRE elements, indicating that most of the *CeqSnRK* genes are involved in ABA signaling transduction pathway. The SA-responsive element (TCA-element) was found in 17 *CeqSnRK* genes, which was also a common cis-acting element.

In addition to *CeqSnRK3.6*/-*3.11*/-*3.16*, cis-elements responding to methyl jasmonate (MeJA) were found in the promoters of the remaining 23 *CeqSnRK* genes. Moreover, elements responding to auxin (AuxRR-core and TGA element) and the gibberellin-responsive element (TATC-box, GARE-motif, and P-box) were also detected in the promoter sequences, with GARE-motif only present in a few *CeqSnRK* genes. Among the cis-acting elements associated with stress, drought (MBS), low temperature (LTR), anaerobic (ARE), and stress response (TC-rich Repeats) elements were found in the promoters of 16, 17, 22, and 9 *CeqSnRK* genes, respectively.

Furthermore, 54 elements partook in plant growth and development were also discovered, among them, the element related to seed-specific regulation (RY-element) was only present in *CeqSnRK3.7* and *CeqSnRK2.1*. The differentiation of the palisade mesophyll cells (HD-Zip1), the meristem expression (CAT-box), and endosperm expression (GCN4-motif) were found in the *CeqSnRK3.16* promoter. In this study, 278 hormone-responsive elements, 124 stress-related response elements, and 54 growth-related elements were discovered in the *CeqSnRK* gene promoters, indicating that the *CeqSnRK* genes may respond to various stresses, as well as be involved in important developmental processes.

### Expression profile of SnRK genes in different tissues

**Fig. 8:**
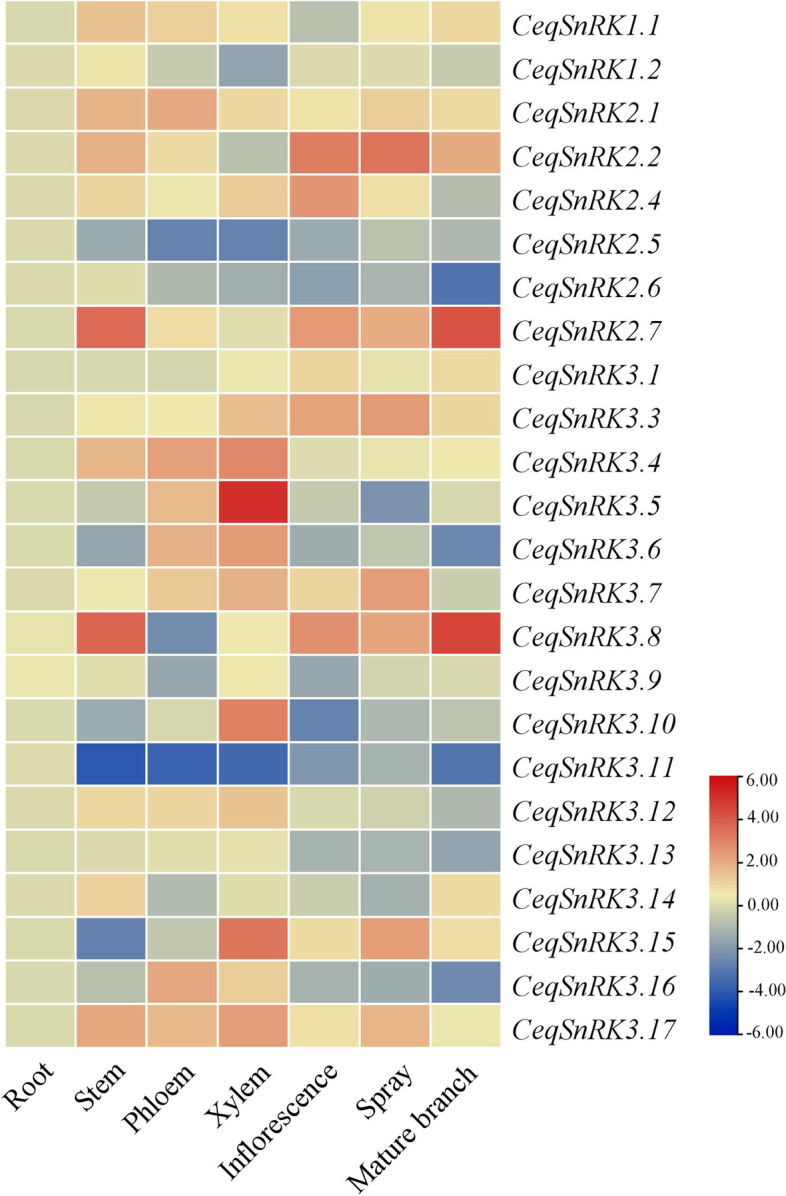
Expression analysis of *CeqSnRK* genes in various tissues. The heatmap shows the hierarchical clustering of 24 *CeqSnRK* genes in different tissues. The color scale on the right-hand side represents log_10_ expression values; white represents low expression, and red indicates a high expression

A heat map was generated using RT-qPCR data from 24 *CeqSnRK* genes, finding that these patterns differed considerably between tissues (Fig. 8). Additionally, we did not detect the expression of *CeqSnRK2.3* or *CeqSnRK3.2*. Most of the genes had low expression levels in roots, except for *CeqSnRK2.5*, *CeqSnRK2.6*, and *CeqSnRK3.11* (Fig. 8). The expression levels of *CeqSnRK3.5* and *CeqSnRK3.11* in xylem, phloem, stem, inflorescence, spray, and mature branches were low, indicating an opposite expression pattern to that in root. Moreover, 7 of 24 *CeqSnRK* genes were highly expressed in xylem, especially *CeqSnRK3.5*, *CeqSnRK3.10*, and *CeqSnRK3.15*. Notably, the genes clustered together in the heat map presented similar expression patterns. For example, *CeqSnRK2.2*, *CeqSnRK3.3*, *CeqSnRK2.4*, and *CeqSnRK3.1* were clustered together and showed lower expression levels in roots, shoots, xylem, and phloem, but higher in inflorescences. Similar patterns were also observed for *CeqSnRK1.1*, *CeqSnRK1.2*, *CeqSnRK2.1*, and *CeqSnRK2.6*.

### CeqSnRK gene expression under salt treatment

**Fig. 9:**
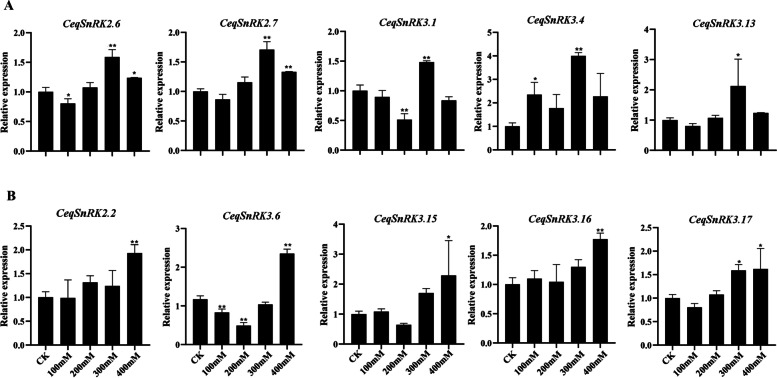
**A** The expression levels of 5 *CeqSnRK* genes were up-regulated and peaked under 300 mM NaCl. **B** The expression levels of 5 *CeqSnRK* genes were up-regulated and peaked under 400 mM NaCl. The *Y*-axis and *X*-axis indicate relative expression levels and salt concentration of stress treatment, respectively. Mean values and standard deviations (SDs) were obtained from three biological and three technical replicates. The error bars indicate standard deviation. **P < 0.01 and *P < 0.05

It was obvious that the expression level of a few *SnRK* genes was up-regulated or down-regulated under diverse NaCl treatments in roots of *C. equisetifolia* (Fig. S3). In particular, the expression levels of 11/24 *CeqSnRK*s were increased following NaCl treatment (Fig. 9); for example, the expression of *CeqSnRK3.4* and *CeqSnRK3.6* peaked at 300 mM and 400 mM salt treatment, respectively (Fig. 9A, B). We obtained similar results for the paralogous *CeqSnRK3.13*/*-3.16*, which were markedly up-regulated in roots at high salinity treatment (300/400 mM) (Fig. S3). However, the expression levels of the paralogous *CeqSnRK1.1*/*-1.2* were strongly down-regulated under high salt treatment (300 and 400 mM).

**Fig. 10:**
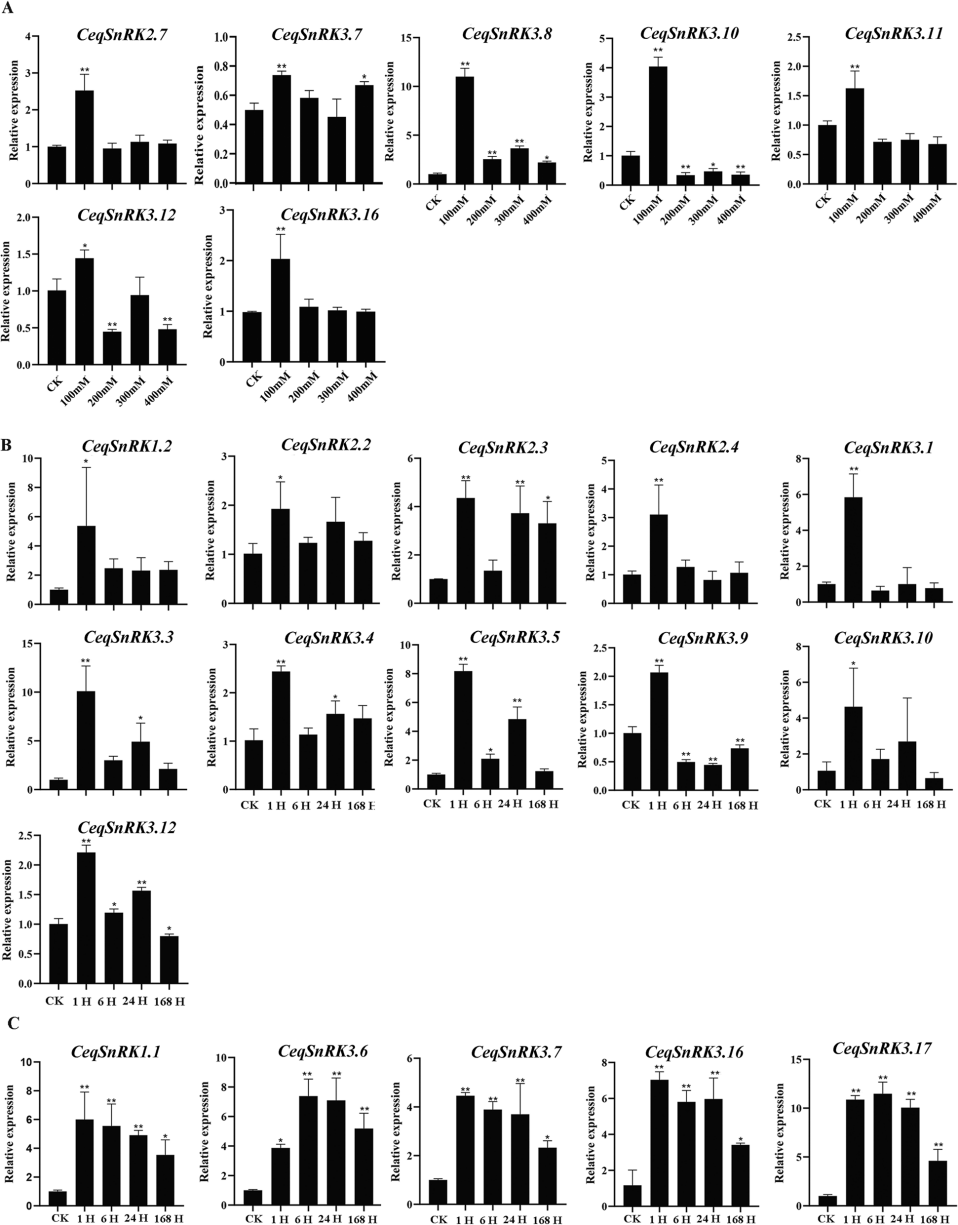
Relative expression of several *CeqSnRK* genes in shoots following NaCl treatment as determined by RT-qPCR. **A** The expression levels of seven *CeqSnRK* genes were up-regulated and peaked under 100 mM NaCl. **B** The expression levels of 11 *CeqSnRK* genes were up-regulated and peaked at 1 h following 200 mM NaCl treatment. **C** The expression levels of 11 *CeqSnRK* genes were up-regulated at different time points following 200 mM NaCl treatment. The *Y*-axis and *X*-axis indicate relative expression levels and the time courses of stress treatments, respectively. Mean values and standard deviations (SDs) were obtained from three biological and three technical replicates. The error bars indicate standard deviation. **P < 0.01 and *P < 0.05

Subsequently, the *CeqSnRK* gene expression profiles in shoots after treatment with different concentrations of NaCl were analyzed (Fig. S4). Of the 24 *CeqSnRK* genes, compared with their expression in the control (non-treated seedlings), 11 were up-regulated under various salt concentrations, 12 were down-regulated, and only *CeqSnRK1.2* did not show any significant change. Of note, the expression levels of seven *CeqSnRK*s were found to be significantly enhanced and reached a maximum at 100 mM after NaCl treatment, followed by a sharp downregulation (Fig. 10A). For example, the expression levels of *CeqSnRK3.8* (more than 11 times) and *CeqSnRK3.10* (about fourfold) were rapidly increased under 100 mM treatment compared with that of the control (Fig. 10A). Moreover, *CeqSnRK1.1*, *CeqSnRK2.4*, and *CeqSnRK3.5* were greatly up-regulated under high concentrations of NaCl (Fig. S4). Interestingly, the expression pattern of *CeqSnRK2.4* was distinctive from that of other *SnRK* genes, mainly showing a trend of first decreasing and then increasing. In other words, the expression of *CeqSnRK2.4* was slightly depressed at 100 mM and then was increased about fivefold under 200 mM. With the further increase in salt concentration, the expression of *CeqSnRK2.4* remained at a high level.

We further analyzed the expression profile of *CeqSnRK* genes after different periods of salt treatments and found 17 genes to be distinctly up-regulated at several time points, while 8 genes were down-regulated or not changed at any time point, compared to the control values (Fig. S5). For example, the expression of 11 genes intensely increased and peaked at 1 h after NaCl treatment and then distinctly decreased at subsequent moments (Fig. 10B). Notably, the expression levels of *CeqSnRK1.1*, *CeqSnRK2.1*, *CeqSnRK3.6*, *CeqSnRK3.7*, *CeqSnRK3.16*, and *CeqSnRK3.17* were higher at each time point under NaCl treatment than in the control (Fig. 10C). Specifically, *CeqSnRK3.16* was drastically up-regulated (sevenfold) at 1 h, and the up-regulation level was maintained at subsequent time points. In contrast, the expression of four *CeqSnRK* genes was significantly descended at each time period. In addition, the effect of diurnal cycle on the experiment was not considered for the time being in the time course analysis of this paper.

### Correlations and co-regulatory networks of CeqSnRK genes

**Fig. 11:**
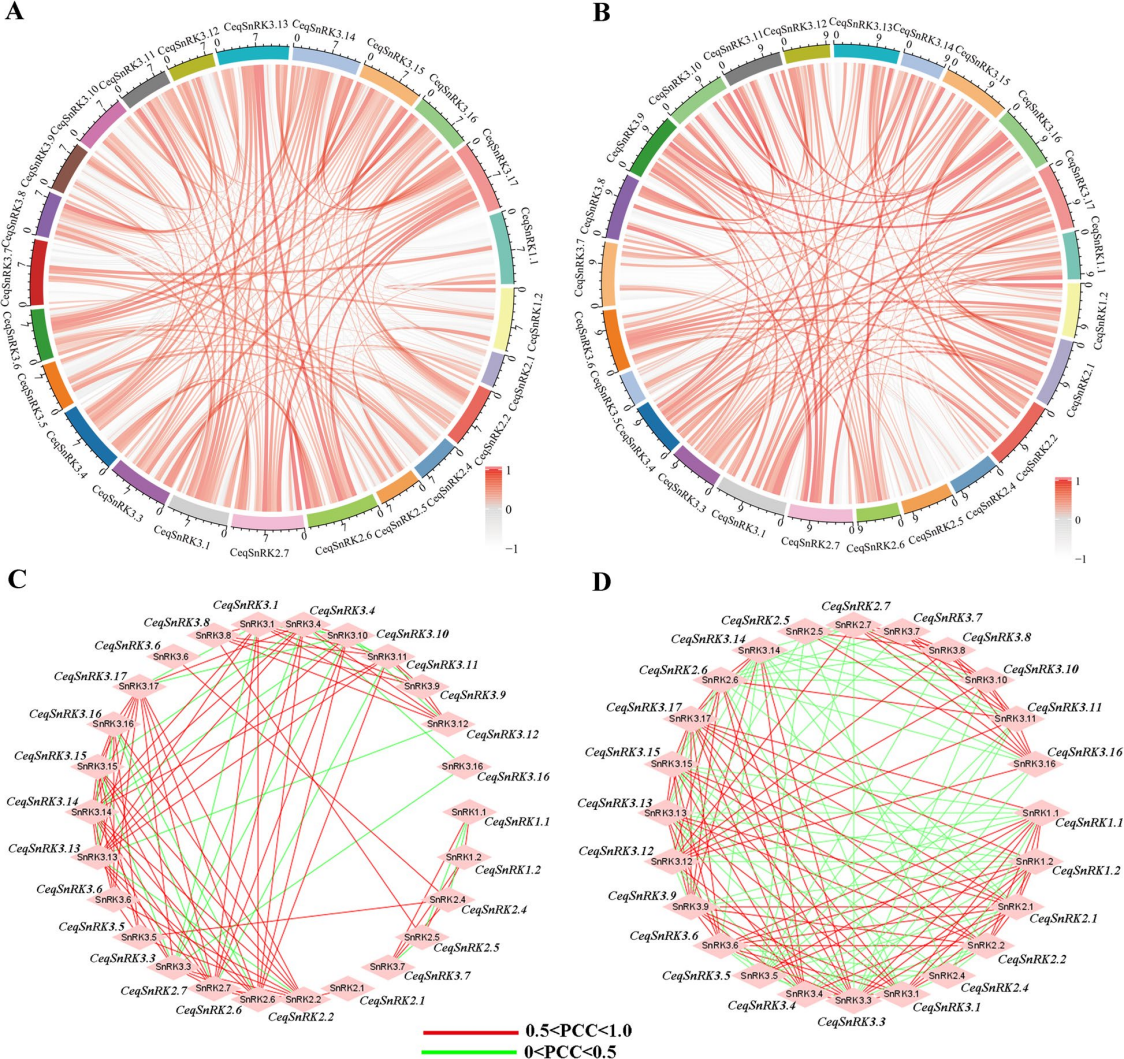
Correlations and co-regulatory networks of *CeqSnRK* genes under stress treatments. **A**, **B** Correlation analysis of *CeqSnRK* genes under NaCl treatment in roots and shoots, respectively. Each correlation is shown by a smooth curve. Red and gray indicate positive and negative correlations, respectively. **C, D** The coregulatory network of *CeqSnRK* genes under NaCl treatment in roots and shoots, respectively. Pearson’s correlation coefficients of co-regulatory gene pairs were considered significant at the 0.05 significance level (*P*-value), and the different correlation levels of the gene pairs are marked by edge lines with different colors

To explore the relationship between the responses of these *CeqSnRK* genes to salt stress, the correlation and co-regulatory networks were established in accordance with the Pearson’s correlation coefficient data for relative expression levels (Fig. 11). For resisting salt pressure, the expression changes of most genes (60 and 57% of genes in roots and stems, respectively) were positively correlated. However, some showed a negative correlation trend. For example, there was a negative correlation between *CeqSnRK3.7* and *CeqSnRK3.17/-3.14/*-*3.13* expression in both roots and shoots (Fig. 11A and B). Moreover, several gene pairs exhibited positive correlations in the root system but negative correlations in shoots. For example, *CeqSnRK2.7* expression was positively correlated with that of *CeqSnRK2.1* and *CeqSnRK2.2* in the root system, but negatively correlated in the shoots.

The correlation coefficient of more than 0.5 in the shoots was significantly higher than that in the roots (Fig. S6), according to the co-regulatory networks associated with salt stresses (Fig. 11C and D). Meanwhile, most of the positive correlations occurred between members belonging to the same subfamily, especially the SnRK3 subfamily. For example, *CeqSnRK3.16*, *CeqSnRK3.15*, and *CeqSnRK3.17* showed significant positive correlations (*P-*value ≤ 0.01 and 0.7 < PCC) under NaCl stress. In addition, *CeqSnRK3.16* expression was positively correlated with that of most *CeqSnRK* genes after salt treatments, suggesting it might respond to salt stress by interacting with other genes. This suggests that gene duplication not only leads to functional divergence but also enhances the synergistic interactions of homologues to help plants adapt to their complex habitats.

## Discussion

According to previous reports, 34, 48, 39, 44, and 60 *SnRK* genes have been identified from *E. grandis* [34], rice [35], *A. thaliana* [12], *B. distachyon* [28], and *Hedychium coronarium* [36], respectively. Various roles of *SnRKs* in plant growth, development, and resistance to biotic and abiotic stresses have been reported, but less is known about the functions of these proteins in *C. equisetifolia*. Hence, we investigated systematically *CeqSnRK* genes by combining bioinformatic analysis and RT-qPCR experiment.

The 26 *CeqSnRK* genes were identified and separated into three subfamilies based on a comprehensive phylogenetic tree. There were 2, 7, and 17 *CeqSnRK* genes in the CeqSnRK1, CeqSnRK2, and CeqSnRK3 subfamilies, respectively. Moreover, different *SnRK* gene subfamilies contained various conserved domains, but the N-terminal protein kinase domain was retained throughout the family. We also discovered that there was a NAF domain in the CeqSnRK3 subfamily at the C terminus (Fig. 2). Based on a previous study, *SnRK3* genes can cooperate with CBLs in a calcium-dependent pattern because of the existence of NAF domain, implying that CeqSnRK3s might respond to stresses by interacting with CBLs [18, 19, 37, 38]. Furthermore, most *CeqSnRK* genes were clustered with the *PtSnRK* rather than the *OsSnRK* genes, implying that *CeqSnRK* and *PtSnRK* genes have a relatively close evolutionary relationship and that there are significant evolutionary differences between the *SnRK* genes in dicots and monocots.

Genetic structural diversity is the main source of multigene family evolution [29, 30]. Therefore, the conserved motifs and gene structure of 26 *CeqSnRK* genes were analyzed. The number and distribution of exon/intron structures differed, but most genes within the same subfamily shared similar gene structures. However, the *CeqSnRK3* subfamily could be subdivided, based on the number of introns, into two kinds, the intron-rich and intron-deficient clades, which is similar to the differentiation of the *HcSnRK3* genes of *H. coronarium* [36]. During evolution, intron-rich populations were inclined to lose introns, thus becoming intron-deficient [39]. Most (9/17) *CeqSnRK3* genes had no intron, suggesting that this subfamily was prone to loss of introns and that the whole family was likely to be more conservative with evolution. We also observed that the most conserved motifs among members of the same subfamily shared certain similar characteristics (Fig. 4), which might give evidence of a closer evolutionary relation within subfamilies. Moreover, motifs 4, 1, 7, and 14 appeared in every *CeqSnRK* gene (Fig. 5A), implying their significance of the function in *CeqSnRKs*. The similarities in structure and motif composition among *SnRK* genes were coincident with our phylogenetic analysis. Meanwhile, the differences among subfamilies suggest that the functions of *SnRK* members are diverse.

Accumulating evidence suggests that gene activity is often relevant with differences in promoter regions, as cis-elements play an important role in controlling gene expression during development and environmental changes [32, 33]. In soybean (*Glycine max*), *GmSnRK2.16* and *GmSnRK2.18* can respond to salt stress due to the presence of two MeJA-responsive elements in their promoters, while *GmSnRK2.6*, which lacks such an element in its promoter region, cannot respond to salt stress [40]. Consistent with this, the expression level in roots of *CeqSnRK3.11*, which lacks a MeJA-responsive element in its promoter (Fig. 7), was not significantly changed under different concentrations of NaCl. Moreover, 24 out of 26 *CeqSnRK* genes contained ABRE cis-regulatory elements related to ABA responsiveness. Accumulating evidence suggests that ABRE-binding protein/ABRE-binding factor (AREB/ABF) can positively regulate plant responses and tolerance, and the SnRK-ABF pathway plays a crucial role in abiotic stress resistance [41–43]. In wheat, *TaSnRK2.9* can interact with *NtABF2* (*N. tabacum*) and up-regulate the expression of *NtABF2* under mannitol (that is, osmotic stress) or NaCl treatment [23]. These results indicate that *SnRK* genes could heighten the resistance to salt though mediating plant hormone signaling.

Gene expression profiles can provide important clues to reveal gene functions [28, 44]. We analyzed the expression of all 26 *CeqSnRK* genes under diverse NaCl treatments. The expression of 11 genes was increased in both roots and shoots with the treatment of NaCl, and among these, *CeqSnRK2.7*, *CeqSnRK3.8*, and *CeqSnRK3.16* were significantly up-regulated. In contrast, *CeqSnRK3.9* was down-regulated in both roots and shoots under different concentrations of NaCl. Analogous expression patterns existed in some paralogous pairs in the same tissue. For example, under high NaCl concentrations (300 and 400 mM), the expression levels of both *CeqSnRK3.13* and *CeqSnRK3.16* were not only significantly up-regulated in roots but similar to control expression levels in shoots (Figs. 9 and 10). Moreover, the expression levels of some *CeqSnRK* genes, such as *CeqSnRK3.5*, *CeqSnRK3.11*, and *CeqSnRK3.12*, were increased in shoots but decreased in roots under salt stress. Similar results were observed in *E. grandis* [34], suggesting that some *SnRK* genes are specifically expressed in shoots. *CeqSnRK3.12*, *EgrSnRK3.9*, and *AtCIPK21*—the latter known to regulate osmotic and salt stress—clustered together [45]. We found that the expression of *CeqSnRK3.12* was obviously increased in shoots under 100 mM NaCl treatment. Thus, *CeqSnRK3.12* may respond to salt stress. Furthermore, most *CeqSnRK* genes were up-regulated at different time points under saline condition in shoots (Fig. S4). Specifically, the expression levels of *CeqSnRK3.6*, *CeqSnRK3.7*, *CeqSnRK3.16*, and *CeqSnRK3.17* were significantly up-regulated at each time period under NaCl treatment compared with the non-treated control (Fig. 10). Previous studies have shown that *AtCIPK24* transports excess intracellular Na^+^ to the extracellular compartment under salt stress [46], thereby increasing the salt tolerance of plants. *CeqSnRK3.13* and *CeqSnRK3.16* were clustered in the same group with *AtCIPK24*, indicating that *CeqSnRK3.13* and *CeqSnRK3.16* might respond to salt stress. Expression of *AtCIPK3* increases the tolerance of *A. thaliana* to high salt concentrations, drought and other stress stimuli [47]. In this study, *CeqSnRK3.7*, which is a homolog of *AtCIPK3*, was strongly up-regulated at all time points under salt treatment, indicating that *CeqSnRK3.7* could play important roles in responses to salt stress. Taken together, our results indicate that most *CeqSnRK* genes were up-regulated to a degree after treating by NaCl, suggesting that *CeqSnRK* genes might be conducive to improving salt tolerance in *C. equisetifolia*.

## Conclusions

*SnRK* genes are involved in a variety of signaling pathways, including responses to biotic and abiotic stresses. In the present study, we identified 26 *CeqSnRK* genes and divided them into three subfamilies on the basis of motif composition and gene structural similarity. Promoter analysis revealed *CeqSnRK* genes involved in plant development and responses to stress and hormones. The expression levels of most *SnRK* genes were induced in shoots and roots under disparate salt treatments. Notably, *CeqSnRK3.16* expression was up-regulated under salt treatment, suggesting that it might respond to salt stress. Moreover, *CeqSnRK3.16* showed significant expression change correlations with multiple genes under salt stress, indicating that it might have synergistic effects with other genes in response to salt stress. Taken together, these results provided a rich theoretical basis for further validation of the functions of *CeqSnRK*s and their roles in salt tolerance.

## Materials and methods

### Identification of SnRK proteins in the C. equisetifolia

Download the whole genome protein sequence of Casuarina from the Casuarina database (http://forestry.fafu.edu.cn/db/Casuarinaceae/) as a local database [36]. The SnRK protein sequences in *A. thaliana* downloaded from the Phytozome database (http://www.phytozome.net/) was used as the target sequence, and then BLAST (E-value-0.5) homology alignment was performed with the local data to obtain the candidate SnRK protein sequences in *C. equisetifolia* [48]. Finally, all candidate *SnRK* genes were manually filtered based on the information from the Pfam database (http://pfam.janelia.org/), the NCBI Conserved Domain database (http://www.ncbi.nlm.nih.gov/Structure/cdd/wrpsb.cgi), and the SMART database (http://smart.embl-heidelberg.de/) [49]. Additionally, detailed resources on *SnRK* genomes in *E. grandis*, *P. trichocarpa*, and rice were obtained from a previous study [34]. Furthermore, the basic physicochemical property parameters of each *SnRK* gene, including information such as open reading frame (ORF) length, molecular weight (MW) and isoelectric point (pI) were explored using the online website Expasy (http://www.expasy.ch/tools/pi_tool.html). The online website WoLP PSORT (https://wolfpsort.hgc.jp/) was used to predict the subcellular localization of the *CeqSnRK* genes.

### Multiple sequence alignment and phylogenetic analyses

All SnRK protein sequences from *A. thaliana*, *C. equisetifolia*, *E. grandis*, *P. trichocarpa*, and rice were aligned using ClustalX 2.11, and a phylogenetic tree was constructed using the neighbor-joining (NJ) method [49, 50] in MEGA 7.0 with a bootstrap value of 1000 [51]. DNAMAN was used to show multiple sequence alignment of *CeqSnRK* genes [36].

### Identification of conserved motifs and analyses of gene and protein structure

The gene structures of exon–intron distribution of *CeqSnRK*s were predicted using the Gene Structure Display Server (GSDS:http://gsds.cbi.pku.edu.ch) based on the coding sequence of each *SnRK* gene and its corresponding genomic DNA sequence. To analyze the conserved motifs of *CeqSnRK* proteins, Multiple Expectation Maximization for Motif Elicitation (MEME) (http://meme.sdsc.edu/meme/itro.html) was used with the following parameters: number of repetition = any; maximum number of motifs = 20; and optimum motif length = 6–200 residues [14, 28]. The 3D structure of each *CeqSnRK* was determined using SWISS-MODEL (https://swissmodel.expasy.org/interactive) [52].

### Ka and Ks analyses of homologous pairs

The paralogs and orthologs were identified on the basis of the way described by Wang et al. [35]. That is, the coding sequences of *SnRK* genes of five species were aligned pairwise by local BLASTN, and the candidate gene pairs of paralogous genes of *C. equisetifolia* and orthologous pairs of *C. equisetifolia* and other four species were initially screened. Afterward, the definition in the previous study was used to further confirm. The synonymous (Ks) and non-synonymous (Ka) substitutions each locus between duplicated genes pairs were calculated using DnaSP v5.0 [53]. To further analyze Ka/Ks values, GraphPad Prism 5 was used to output sliding window graphs for analysis.

### Cis-elements in the promoter regions of CeqSnRK genes

The promoter region, 2000 bp upstream of the translation start of each *CeqSnRK* gene, was searched using all Casuarina whole genome sequences and GFF files. The subsequent prediction and analysis of cis-acting elements were completed using the online site PlantCARE (http://bioinformatics.psb.ugent.be/webtools/plantcare/html/) [54].

### Plant materials and stress treatments

Clone A8 of *C. equisetifolia* is widely used for coastal shelterbelt construction in Guangdong province. Different tissues (root、stem、phloem、xylem、inflorescence、spray and mature branch) were taken from 10-year-old clone A8 in our nursery. Then, young branchlets of A8 were collected and vegetatively propagated to obtain experiment material for this study. After hydroponically rooting, clone A8 was transferred to a greenhouse and cultured for 3 months until the seedlings reached 60–70 cm in height. Subsequently, two salt treatments were included, one with 200 mM NaCl for 0, 1, 6, 24 and 168 h and the other with different salt concentrations (0, 100, 200, 300, and 400 mM) for 24 h. Plant samples (roots and shoots) were taken after watering with the various NaCl solution. All collected samples were immediately frozen with liquid nitrogen and transferred to − 80℃ for storage until RNA extraction.

### RNA extraction and RT-qPCR analysis

Total RNA was isolated from each sample following the protocol as described previously and examined using 1% agarose gel electrophoresis and a NanoDrop™ One/OneC (Thermo Fisher Scientific, USA) [34]. According to the instructions, the first-strand cDNA synthesis and RT-qPCR were completed using PrimerScript RT MasterMix (Takara, Tokyo, Japan) and Swiss-made LightCycler480 II Real-Time PCR system, respectively. The loading system was: 10 μL Premix Ex Taq II, 2 μL cDNA template, 0.4 μL ROX Reference Dye (50X), 6 μL sterile water, and 0.8 μL forward and reverse primers each. qPCR reaction conditions were as follows: 95 ℃ for 30 s, followed by 40 cycles of denaturation at 95 ℃ for 5 s, and annealing at 55–60 ℃ for 34 s [54]. Based on literature reports, using *EF1*α as the reference gene, primers were designed using Primer 6 software [55] and then sent to the company (Ruibo, Guangzhou) for synthesis. The primer sequence was detailed in Table S8. Three biological and technical replicates were performed for each real-time PCR reaction [35]. The experimental data were processed by the 2^−ΔΔct^ method [56], and the significant difference in the data was calculated by using IBM SPSS Statistics 25 software. Statistical analysis and graphing of gene expression patterns were completed using GraphPad 8 [57]. The obtained data were log_2_-transformed and visualized as a heatmap using TBtools [58].

### Pearson’s correlation analyses

Pearson’s correlation coefficients of *CeqSnRK* gene expression levels were calculated and visualized using R (https://www.r-project.org) based on the RT-qPCR results [59]. Pairs of genes that were positively correlated with each other were collected for a gene coregulatory network analysis. The co-expression networks were graphically visualized using Cytoscape based on the Pearson’s correlation coefficients of these gene pairs [9].

## Supplementary Information

**Additional file 1:** **Figure S1**. Tertiary structures of the CeqSnRKproteins**. **Protein models were obtained using the SWISS-MODEL online server.

**Additional file 2:** **Figure S2**.Ka/Ks ratios of orthologs.Left image represents orthologs of *CeqSnRK* with *EgrSnRK* and *PtSnRK*.Right image represents orthologs of *CeqSnRK*with *AtSnRK* and *OsSnRK*.

**Additional file 3:** **Figure S3**.Relative expression of *CeqSnRK* genes in roots following different NaCltreatments as determined by RT-qPCR. The *Y*-axis and *X*-axisindicate relative expression levels and salt concentration of stress treatment,respectively. Mean values and standard deviations (SDs) were obtained from threebiological and three technical replicates. The error bars indicate standarddeviation. **P < 0.01 and *P < 0.05. 

**Additional file 4:** **Figure S4**.Relative expression of *CeqSnRK* genes in shoots following different NaCltreatments as determined by RT-qPCR. The *Y*-axis and *X*-axisindicate relative expression levels and salt concentration of stress treatment,respectively. Mean values and standard deviations (SDs) were obtained fromthree biological and three technical replicates. The error bars indicate standarddeviation. **P < 0.01 and *P < 0.05.

**Additional file 5:** **Figure S5**.Relative expression of 25 selected *CeqSnRK*genes in shoots at different times following NaCl treatment as determined byRT-qPCR.The *Y*-axis and *X*-axis indicaterelative expression levels and the time course of stress treatment,respectively. Mean values and standard deviations (SDs) were obtained fromthree biological and three technical replicates. The error bars indicatestandard deviation. **P < 0.01 and *P < 0.05.

**Additional file 6:** **Figure S6**. Correlation matrixof expression among the *CeqSnRK* genesunder NaCl treatment. Correlation analysis of the expression of *CeqSnRK* genes under NaCl treatment inroots (left) and shoots (right). Correlations are indicated by the size andcolor of circles. * and ** represent correlations with *P*-value ≤ 0.05and *P*-value ≤ 0.01, respectively. 

**Additional file 7:** **Table S1 **Details of the identified *CeqSnRK* genes. **Table S2.** The prediction of subcellularlocalization. **Table S3. **Details of *SnRK*genes from *Arabidopsis*, rice, grapevine, and *Populous trichocarpa*.**Table S4. **Detailed information for the 20 motifs in the SnRK proteins of *Casuarina equisetifolia*. **Table S5. **Ka/Ks ratios of paralogous andorthologous gene pairs. **Table S6. **Promoteranalysis of the *CeqSnRK* genes. **TableS7. **Promoter analysis of the *CeqSnRK* genes. **Table S8. **List of primer sequences used for RT-qPCRanalysis of the *CeqSnRK* genes.

## Data Availability

The genome sequences of *E. grandis* were downloaded from Phytozome database (https://data.jgi.doe.gov/refine-download/phytozome?genome_id=297&expanded=Phytozome-297); the genome sequences of *P. trichocarpa* were downloaded from Phytozome database (https://data.jgi.doe.gov/refine-download/phytozome?genome_id=210&expanded=Phytozome-210); and the genome sequences of *A. thaliana* were downloaded from Phytozome database (https://data.jgi.doe.gov/refine-download/phytozome?genome_id=167&expanded=Phytozome-167). Rice protein sequences and cDNA sequences was provided by the Rice Annotation Project (RAP) (https://rapdb.dna.affrc.go.jp/download/irgsp1.html). The Casuarina genome data were downloaded from online website (http://forestry.fafu.edu.cn/db/Casuarinaceae/). The datasets supporting the results of this article are included in the article and Additional files.

## Abbreviations

Ka: Number of non-synonymous substitutions per non-synonymous site
Ks: Number of synonymous substitutions per synonymous site
RT-qPCR: Quantitative real‑time PCR
SNF1: Sucrose nonfermenting 1
CIPKs: Calcineurin B-like protein-interacting protein kinases
CBL: Calcineurin B-like protein
SOS: Salt overly sensitive
NJ: Neighbor-joining
