# Supplementary figures and images for: Comprehensive identification and expression analyses of the SnRK gene family in Casuarina equisetifolia in response to salt stress
Source: BMC Plant Biol. 2022 Dec 9;22:572. doi: 10.1186/s12870-022-03961-7 (PMC9733041; doi:10.1186/s12870-022-03961-7)

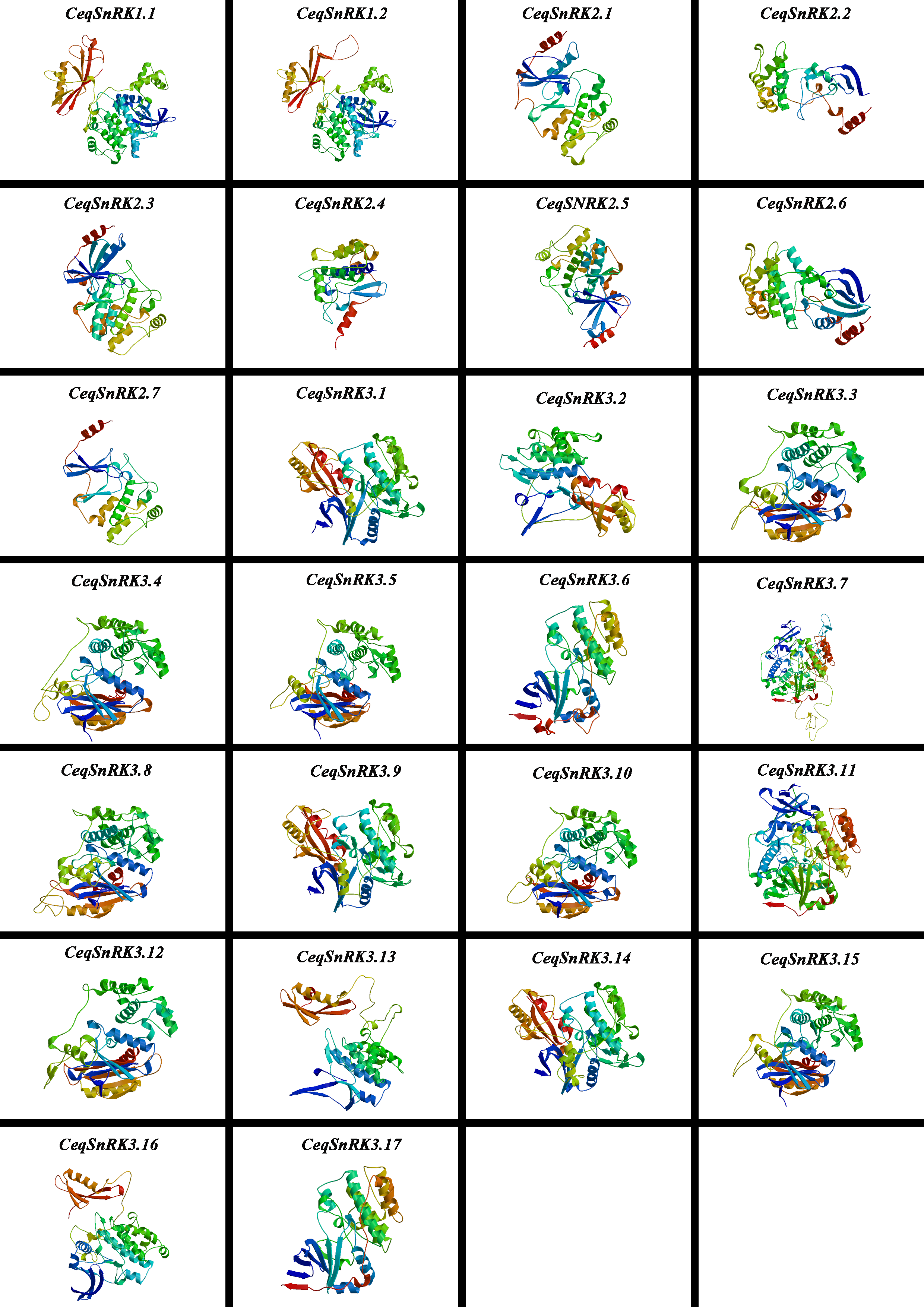

Supplement: Supplementary file 1 — Additional file 1: Figure S1. Tertiary structures of the CeqSnRKproteins. Protein models were obtained using the SWISS-MODEL online server. [file 12870_2022_3961_MOESM1_ESM.tif]

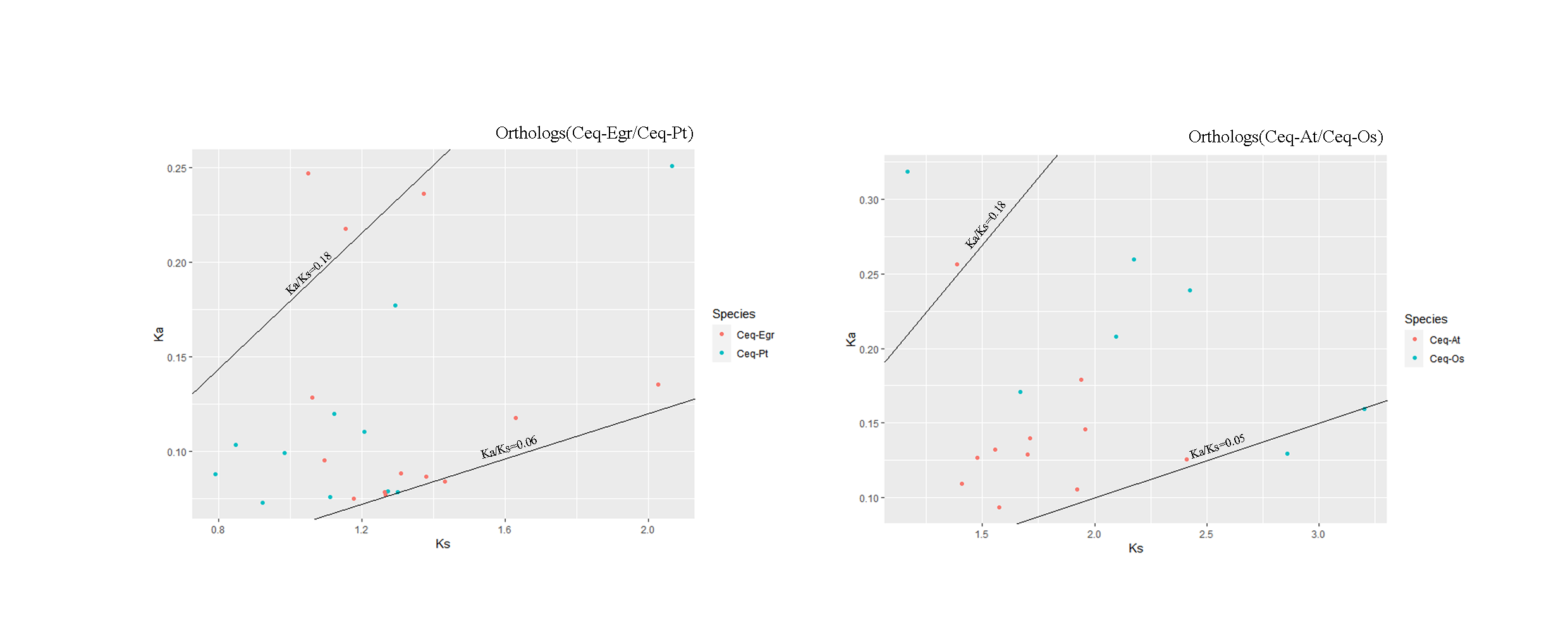

Supplement: Supplementary file 2 — Additional file 2: Figure S2.Ka/Ks ratios of orthologs.Left image represents orthologs of CeqSnRK with EgrSnRK and PtSnRK.Right image represents orthologs of CeqSnRKwith AtSnRK and OsSnRK. [file 12870_2022_3961_MOESM2_ESM.tif]

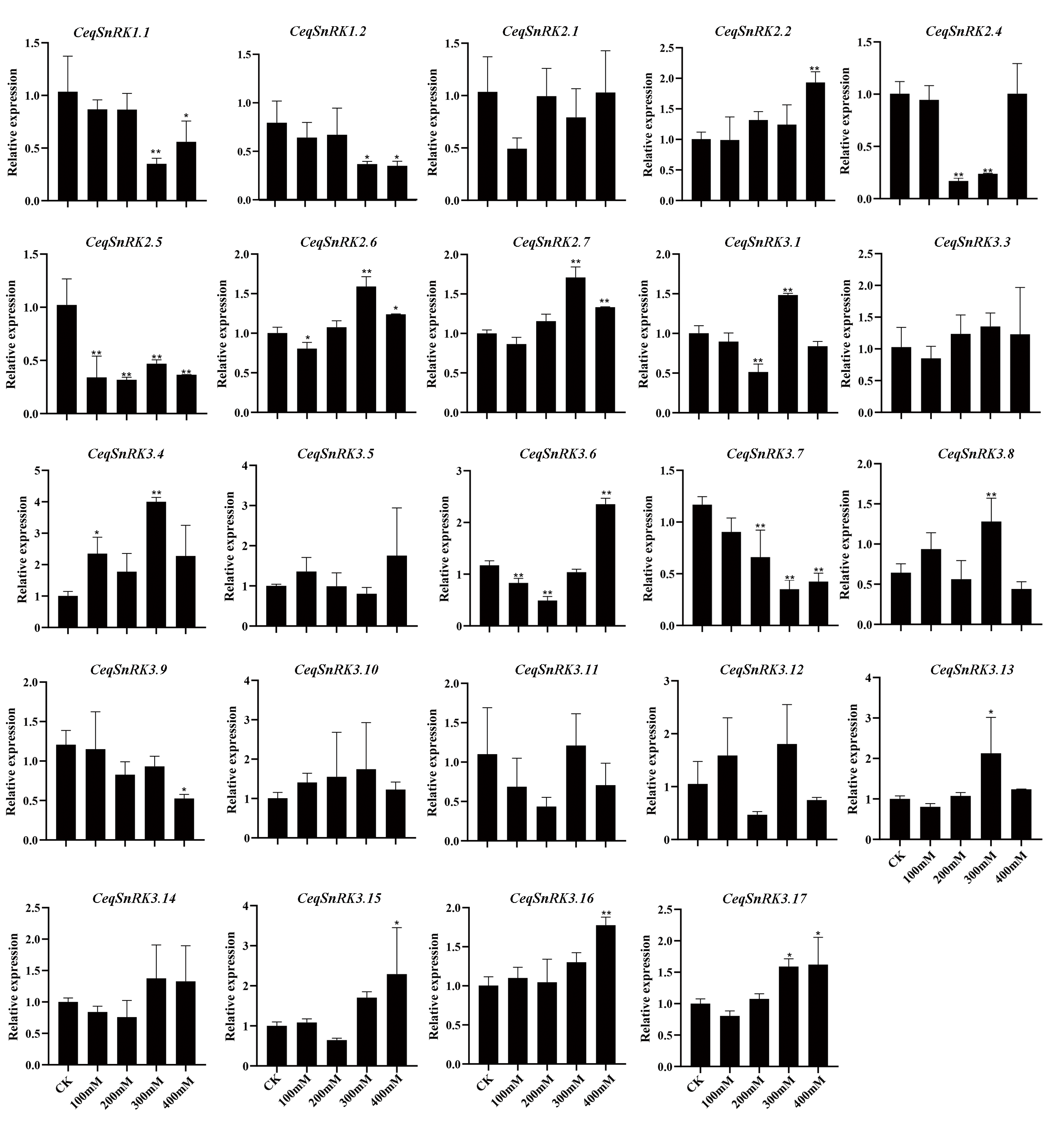

Supplement: Supplementary file 3 — Additional file 3: Figure S3.Relative expression of CeqSnRK genes in roots following different NaCltreatments as determined by RT-qPCR. The Y-axis and X-axisindicate relative expression levels and salt concentration of stress treatment,respectively. Mean values and standard deviations (SDs) were obtained from threebiological and three technical replicates. The error bars indicate standarddeviation. **P < 0.01 and *P < 0.05. [file 12870_2022_3961_MOESM3_ESM.tif]

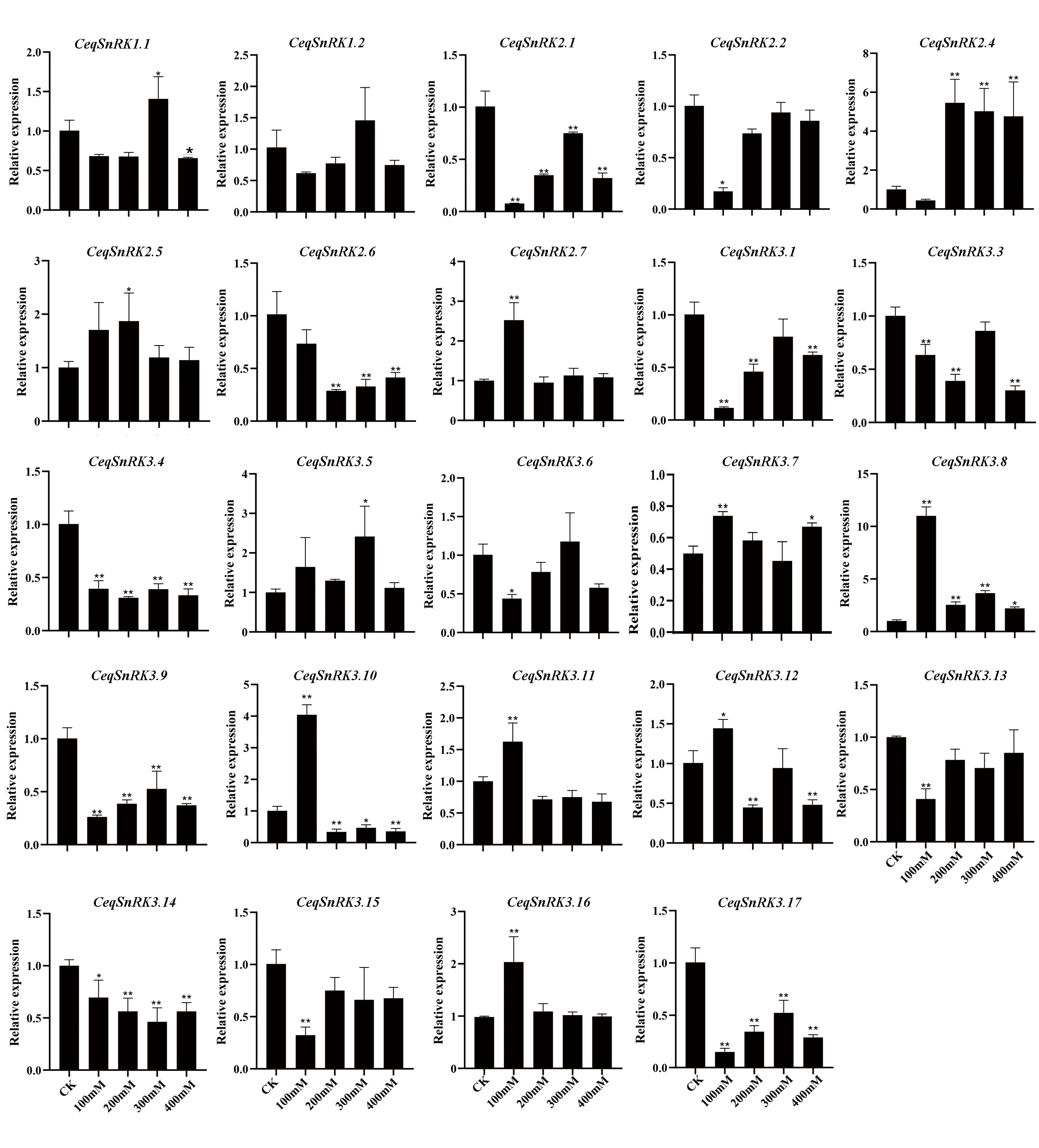

Supplement: Supplementary file 4 — Additional file 4: Figure S4.Relative expression of CeqSnRK genes in shoots following different NaCltreatments as determined by RT-qPCR. The Y-axis and X-axisindicate relative expression levels and salt concentration of stress treatment,respectively. Mean values and standard deviations (SDs) were obtained fromthree biological and three technical replicates. The error bars indicate standarddeviation. **P < 0.01 and *P < 0.05. [file 12870_2022_3961_MOESM4_ESM.tif]

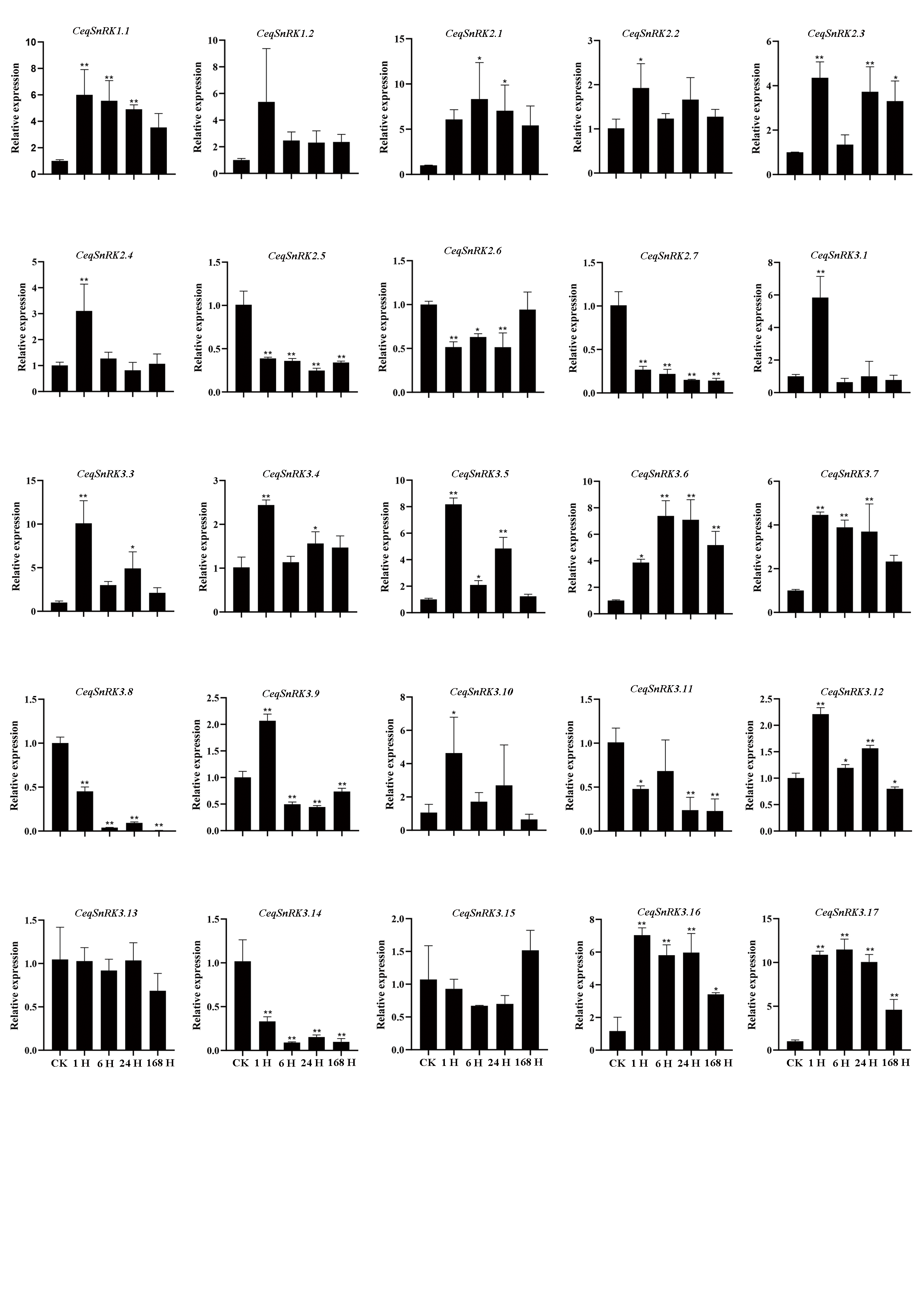

Supplement: Supplementary file 5 — Additional file 5: Figure S5.Relative expression of 25 selected CeqSnRKgenes in shoots at different times following NaCl treatment as determined byRT-qPCR.The Y-axis and X-axis indicaterelative expression levels and the time course of stress treatment,respectively. Mean values and standard deviations (SDs) were obtained fromthree biological and three technical replicates. The error bars indicatestandard deviation. **P < 0.01 and *P < 0.05. [file 12870_2022_3961_MOESM5_ESM.tif]

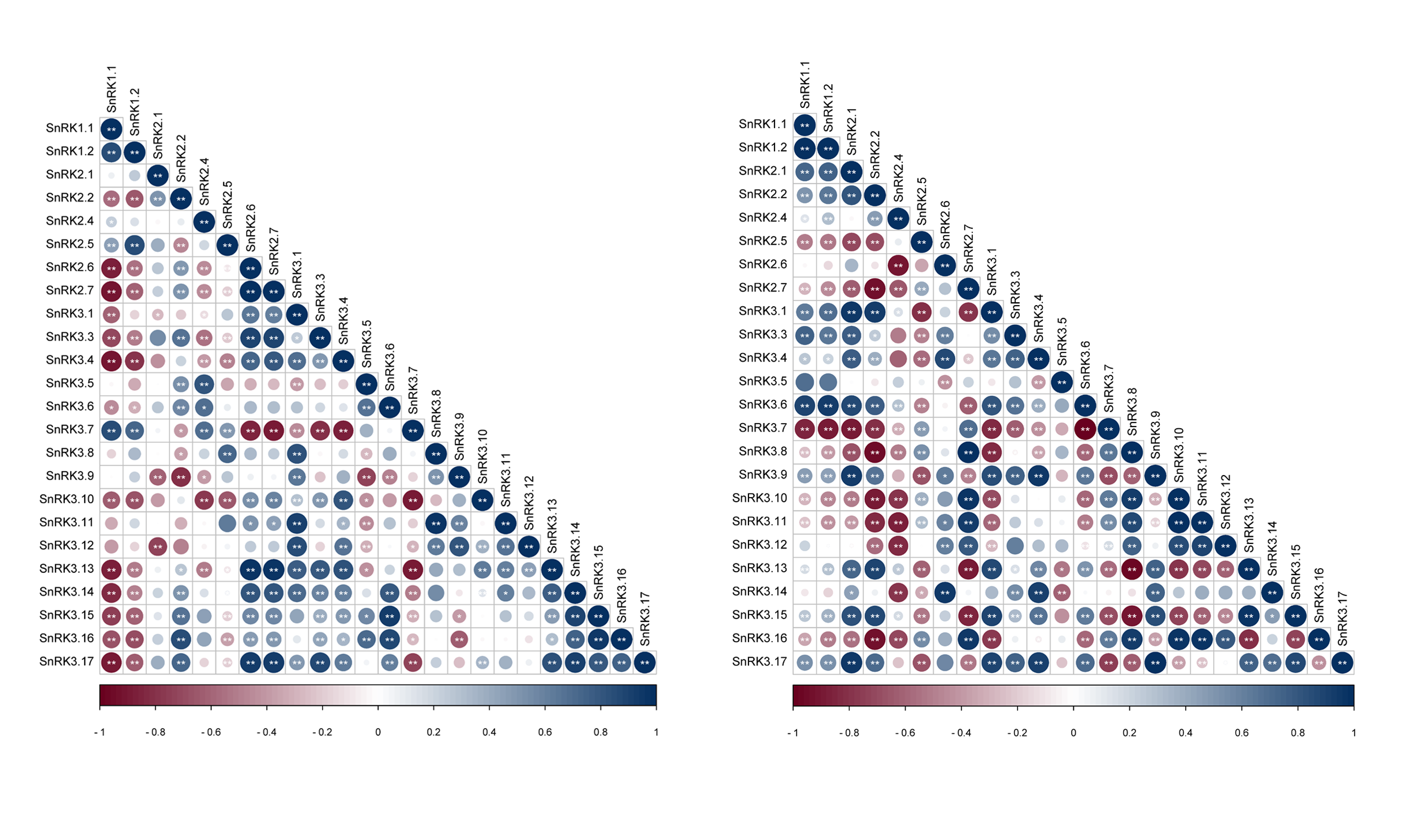

Supplement: Supplementary file 6 — Additional file 6: Figure S6. Correlation matrixof expression among the CeqSnRK genesunder NaCl treatment. Correlation analysis of the expression of CeqSnRK genes under NaCl treatment inroots (left) and shoots (right). Correlations are indicated by the size andcolor of circles. * and ** represent correlations with P-value ≤ 0.05and P-value ≤ 0.01, respectively. [file 12870_2022_3961_MOESM6_ESM.tif]
